# Discordance Between Social Vulnerability and Cancer-Related Mortality in Border Counties - A Letter to the Editor Regarding “Local Social Vulnerability as a Predictor for Cancer-Related Mortality Among US Counties”

**DOI:** 10.1093/oncolo/oyad271

**Published:** 2023-09-28

**Authors:** Michael LaPelusa, Fernando Cristobal Diaz, Patricia Mae G Santos, Haydeé Verduzco-Aguirre, Enrique Soto-Perez-de-Celis

**Affiliations:** Division of Cancer Medicine, MD Anderson Cancer Center, Houston, TX, USA; Lineberger Comprehensive Cancer Center, University of North Carolina – Chapel Hill, Chapel Hill, NC, USA; Department of Radiation Oncology, Memorial Sloan Kettering Cancer Center, New York, NY, USA; Department of Hemato-Oncology, Instituto Nacional de Ciencias Médicas y Nutrición Salvador Zubirán, Mexico City, Mexico; Department of Geriatrics, Instituto Nacional de Ciencias Médicas y Nutrición Salvador Zubirán, Mexico City, Mexico

## Abstract

This letter to the editor remarks on results of a recently published study and highlights the need for multinational and interinstitutional registries so health departments in the US and Latin American countries can accurately capture cancer incidence and mortality data.

In the Brief Communication titled “Local Social Vulnerability as a Predictor for Cancer-Related Mortality Among US Counties” by Chen et al,^1^ the authors showed how more socially vulnerable United States (US) counties experience a higher age-adjusted cancer mortality rate. In Fig. 1A and 1B of their article, an obvious discordance between cancer mortality and social vulnerability index (SVI) exists among counties along the US southern border, similar to what has been reported previously.^2^

US border counties are predominantly composed of Hispanic communities, in which English is not individuals’ primary language, who often travel to Mexico to receive healthcare.^3-5^ People living in US border counties, particularly Texas, have uninsurance rates as high as 47%.^6,7^ US border counties also have a low median household income, as evidenced by Texas’ Starr County, whose median household income in 2018 was $29 294, less than half that of the average Texas county.^8^ These socioeconomic factors contribute to the border region’s high SVI, an index created by the Centers for Disease Control and Prevention, which was used by Chen et al. Exacerbating the high SVI observed in US border counties is a low rate of cancer screening utilization, which is associated with increased cancer mortality in some tumor types.^9^ In 2014, 6 of the 20 US counties with the lowest percentage of the population being up to date with colorectal cancer screening were Texas border counties.^10^ It is unclear why US border counties have a low cancer mortality rate, despite these socioeconomic and public health issues.

One potential explanation for this phenomenon is the so-called “salmon bias effect,” which purports patients return to their country of origin or where their extended family resides when they receive a diagnosis of advanced cancer.^11^ Subsequently, the outcomes of individuals who opt to seek care in their home countries may not be captured in the death statistics of their county, state, or country of residence, resulting in inaccurate cancer mortality registration. Given the high proportion of individuals in US border counties who travel across the border for healthcare, this effect may explain the low cancer mortality rate along the US southern border.

A clear need exists for creating multinational and interinstitutional registries so health departments in the US and Latin American countries can accurately capture cancer incidence and mortality data. These registries could help researchers better delineate the true impact of cancer mortality on communities with a high population of individuals who migrate to and from the US, such as US border counties, so policymakers can deliver the desperately needed resources this often-overlooked area deserves.
